# Use of a public-private partnership in malaria elimination efforts in Sri Lanka; a case study

**DOI:** 10.1186/s12913-018-3008-y

**Published:** 2018-03-23

**Authors:** Deepika Fernando, Pandu Wijeyaratne, Rajitha Wickremasinghe, Rabindra R. Abeyasinghe, Gawrie N. L. Galappaththy, Renu Wickremasinghe, M. Hapugoda, W. A. Abeywickrema, Chaturaka Rodrigo

**Affiliations:** 1 Department of Parasitology, Faculty of Medicine, University of Colombo, Colombo, Sri Lanka; 2 Tropical and Environmental Diseases and Health Associates, No. 3 Elibank Road, Colombo, Sri Lanka; 3 Department of Public Health, Faculty of Medicine, University of Kelaniya, Kelaniya, Sri Lanka; 4 Regional Office for the Western Pacific, World Health Organization, Manila, Philippines; 5 World Health Organization, Hanoi, Vietnam; 6 Department of Parasitology, Faculty of Medical Sciences, University of Sri Jayewardenepura, Nugegoda, Sri Lanka; 7 Department of Parasitology, Faculty of Medicine, University of Kelaniya, Kelaniya, Sri Lanka; 8 Department of Clinical Medicine, Faculty of Medicine, University of Colombo, Colombo, Sri Lanka

**Keywords:** Malaria, Sri Lanka, Elimination, Surveillance

## Abstract

**Background:**

In special circumstances, establishing public private partnerships for malaria elimination may achieve targets faster than the state sector acting by itself. Following the end of the separatist war in Sri Lanka in 2009, the Anti Malaria Campaign (AMC) of Sri Lanka intensified malaria surveillance jointly with a private sector partner, Tropical and Environmental Diseases and Health Associates Private Limited (TEDHA) with a view to achieving malaria elimination targets by 2014.

**Methods:**

This is a case study on how public private partnerships can be effectively utilized to achieve malaria elimination goals. TEDHA established 50 Malaria Diagnostic Laboratories and 17 entomology surveillance sentinel sites in consultation with the AMC in areas difficult to access by government officials (five districts in two provinces affected by war).

**Results:**

TEDHA screened 994,448 individuals for malaria, of which 243,867 were screened at mobile malaria clinics as compared to 1,102,054 screened by the AMC. Nine malaria positives were diagnosed by TEDHA, while the AMC diagnosed 103 malaria cases in the same districts in parallel. Over 13,000 entomological activity days were completed. Relevant information was shared with AMC and the data recorded in the health information system.

**Conclusions:**

A successful public-private partnership model for malaria elimination was initiated at a time when the health system was in disarray in war ravaged areas of Sri Lanka. This ensured a high annual blood examination rate and screening of vulnerable people in receptive areas. These were important for certification of malaria-free status which Sri Lanka eventually received in 2016.

## Background

In May 2009, a 30 year old separatist conflict in the Northern and Eastern provinces of Sri Lanka ended. From 2000 to 2008, of the 335,278 malaria cases reported throughout the country, approximately 70% was from these two provinces [1, 2]. At the time of the conflict, displacement of large populations and poor access to diagnostic facilities delayed treatment for malaria patients. Organized malaria control activities, though few in number, continued with the support of government health care staff, security forces, international and local Non-Governmental Organizations (NGOs) and most importantly, the Anti Malaria Campaign (AMC) which is the dedicated organization of the Ministry of Health to control malaria in Sri Lanka [3].

With cessation of hostilities, the challenge for the government was to develop and strengthen the entire health system in the Northern and Eastern Provinces of Sri Lanka which had been severely affected by the conflict. Malaria surveillance in these areas in 2009 was limited to the larger hospitals that had facilities for microscopy. The infrastructure was poor as hospitals lacked basic equipment and facilities including running water and electricity. There was a dearth of trained personnel in these areas. In comparison to non-conflict districts where routine entomological services were being carried out, in the Northern and Eastern provinces, entomological surveillance was almost non-existent.

The national and provincial governments had limited capacity to uplift the services in these areas within a short period of time. At this juncture, the AMC worked on the hypothesis that a private public partnership would be a better option in achieving the malaria elimination targets in these provinces. It decided to engage the assistance of Tropical and Environmental Diseases and Health Associates Private Limited (TEDHA), a private sector partner, to enhance prevention, and intensify surveillance and control of malaria with a view to achieving elimination targets by 2014 [4]. TEDHA was incorporated as an organization in 2004 just after the Asian Tsunami with a vision of preventing communicable diseases at the regional and global level. TEDHA comprised a core group of health professionals specialized in varied fields ranging from epidemiology, parasitology, entomology, logistics, IT, finance and procurement, who had experience in community level health programmes in Uganda, Kenya, Bangladesh, Nepal and Sri Lanka [5]. Three members of the team who are also co-authors of this manuscript were appointed by the Director General of Health Services as members of the Technical Support Group of the AMC for the malaria elimination programme. TEDHA developed a model for intensified surveillance jointly with the AMC to complement the national malaria control programme in a public-private partnership, rather than create a parallel system, in five districts of the two provinces affected by the conflict. This manuscript discusses the case study of public-private partnership (TEDHA as a partner to the National Anti-Malaria Campaign) during 2010–2014 that eventually helped the country to be certified as “malaria-free” by WHO in September 2016. This case study will be useful for other countries with conflicts and hard-to-reach communities to implement malaria elimination measures.

## Methods

This is a case study on using private-public partnerships in malaria elimination efforts under special circumstances. The public component of this partnership, the Anti Malaria Campaign of Sri Lanka has existed for over 100 years and has been discussed in many publications previously [6]. This description will predominantly concentrate on the private sector entity that entered into a partnership with the AMC.

### TEDHA implementation plan and project management

The implementation plan for TEDHA was conceived to accommodate the needs of the malaria elimination efforts of the country as well as the performance based funding model of the Global Fund. The approach towards intensified surveillance entailed the following activities in the designated geographical areas: building malaria surveillance capacity through recruitment and training of staff to be stationed at government health care facilities; tracking populations, environmental changes and changes in vector densities; enhancing awareness’ advocacy; and complementing existing government mechanisms for malaria elimination. The project management plan was designed such that the programme director oversees the functions to ensure smooth and successful implementation of the malaria surveillance program from the TEDHA head office located in Sri Lanka’s commercial capital, Colombo. The geographic areas for surveillance were assigned by the AMC, in accordance with time-bound targets set by the Global Fund. In order to carry out the surveillance plan effectively at the field level, a district office (DO) was established in each of the five districts TEDHA was operating (Eastern Province: Ampara, Trincomalee and Batticaloa: Northern Province: Mannar and Killinochchi); in addition a sub-district office was established in Ampara district due to its large size. Each DO was managed by a team leader, a retired staff member of the AMC, who was responsible for the overall management of activities in the district with the support of an Administrative Assistant.

### TEDHA’s surveillance approach

At the commencement of surveillance in May–June 2009, the district administrative system was functioning to ensure the infrastructure needed for parasitological and entomological surveillance was in place. In the five districts, the sites for Malaria Diagnostic Laboratories (MDLs) and for entomological surveillance were mutually agreed upon by TEDHA and the AMC. Prior to the onset of the programme, each hospital was visited by TEDHA consultants to identify space to establish the MDL. Recruitment of technical personnel took place after the establishment of the District offices, MDLs and entomology sentinel sites. The number of staff to be recruited and trained in technical activities related to parasitology and entomology, and their subsequent designations were identified.

A curriculum for training technical staff was developed by the consultants of TEDHA and the AMC in Parasitology, Epidemiology, Public Health and Entomology and technical staff were given a six-week training on malaria diagnosis and entomological techniques. A further field training of 2–4 weeks was provided under the supervision of the respective Regional Malaria Officer (RMO) after successful completion of the course which was assessed by an end of course examination. Technical staff that had finished high school and who obtained the highest marks at the assessments were appointed as officers in charge of the district and were located in the district office.

Each entomology team had eight members one vector surveillance assistant, two field vector surveillance assistants and five vector collecting assistants which was similar to the entomology teams of the AMC. Local houses were rented to accommodate the entomological teams. Pedal bicycles and a three-wheeled vehicle were provided to each entomology sentinel site.

### Supervision and monitoring

Field visits to each district were done once a month and the work of fever and parasitology staff were reviewed. If the performance of any member of the technical staff was unsatisfactory, they were provided refresher training. TEDHA recruited a Technical Resource Person with over 30 years experience as a senior Technical Officer in a Parasitology laboratory of a University for quality assurance of microscopy. For entomology, two entomological assistants from the AMC were recruited to carry out technical monitoring. Entomology re-training took place at the sentinel sites.

### Fever and parasitological surveillance

Three malaria surveillance techniques are practiced in Sri Lanka, namely, activated passive case detection (APCD), passive case detection (PCD) and active case detection (ACD). TEDHA carried out PCD and APCD at the MDLs and a report was issued to the patient for each test. The tests were provided free of charge. Positive patients were immediately notified to the RMO and AMC Headquarters (AMC HQ) and treated according to national treatment guidelines by AMC staff. ACD was based on screening pre-identified populations irrespective of whether they had fever or not in the past 2 weeks. This was carried out through Mobile Malaria Clinics (MMC’s) and included high risk populations, pregnant women visiting antenatal clinics and populations resident in close proximity to hospital surveillance sites by home visits. The process of carrying out MMCs is described elsewhere [7].

A quality assurance system was established for microscopy in collaboration with the Central Parasitology Laboratory of the AMC. Ten percent of negative slides and all positive slides were cross-checked by experienced microscopists of the AMC and technical officers of Departments of Parasitology in Faculties of Medicine located in and around Colombo. Data recording forms were adapted from those used by the AMC which enabled the merging of TEDHA data with AMC data.

### Entomological surveillance

Seventeen entomological surveillance sentinel sites were set up in the five districts based on different ecological settings, population density and area. Surveillance was conducted in four localities in each sentinel site (one locality per week and repeated every month). In addition, surveillance was conducted in and around where cases were reported as part of the case investigation process.

Entomological surveillance was monitored in terms of entomological days, defined as conducting at least one entomological surveillance activity per day. Techniques for adult mosquito surveillance was the same used by AMC and included cattle baited net and hut trap collections, exit trap collections, insecticide spray sheet collections, and hand collections [4, 8]. Other activities done at regular intervals included: larval surveys to estimate larval densities; insecticide bioassay tests for indoor residual spraying and long lasting insecticide treated nets; and insecticide susceptibility tests using wild caught mosquitoes [8]. Entomological surveillance data were recorded in forms which were adapted from those used by the AMC. Data, especially when the principal vector *An.culicifacies* was detected were immediately channeled to AMC to guide programme decisions on vector control activities. Quality control and monitoring of the teams were carried out by trained entomological field managers and their assistants under the guidance of the entomology consultants of TEDHA and entomological staff of the RMO’s.

### Community mobilization

Community Mobilization Officers were recruited and trained to carry out advocacy on the programme and to engage with the community. They raised public awareness regarding malaria prevention, control and treatment within the communities and got them involved in the surveillance process. It was envisaged that this would encourage people to visit the malaria diagnostic laboratories of TEDHA and AMC to get tested for malaria when they had fever and participate in mobile malaria clinics.

Community mobilization officers also informed private health care providers in the area including hospitals, clinics and general practitioners of TEDHA activities and requested them to refer suspected malaria cases to the MDL for testing which was provided free of charge.

### Information flow, management and follow up of malaria positives cases

**Fig. 1:**
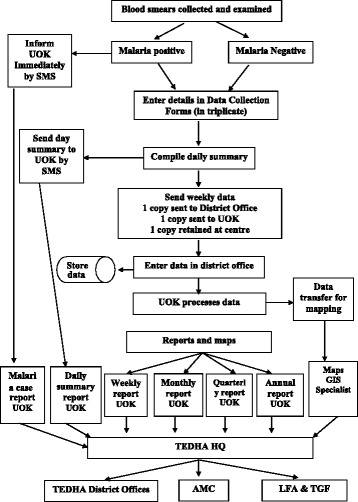
Data flow for fever and parasitological surveillance of the TEDHA surveillance system

**Fig. 2:**
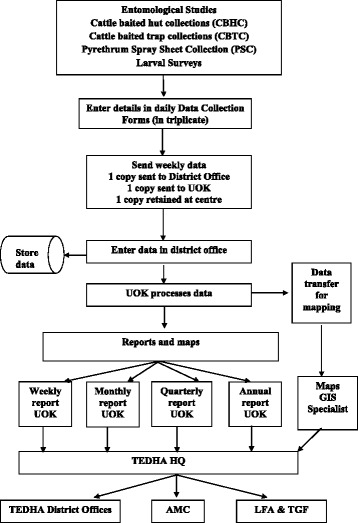
Data flow for entomology

TEDHA was committed to sharing information with AMC HQ and Regional Malaria Offices. The information flow of the TEDHA surveillance systems are depicted in Figs. 1 and 2. TEDHA consultants, RMO of the district and the AMC headquarters were informed by telephone in the event a malaria infection was detected. Treatment was initiated by the AMC as per national malaria treatment guidelines. TEDHA assisted the RMOs in following up cases to ensure complete and radical parasitological cure. Entomological information was shared with the RMOs to initiate necessary vector control activities which were under the purview of the AMC.

An extensive Geographical Information System (GIS) was developed and integrated with AMC as the programme evolved to collate spatial and temporal information of the areas under TEDHA surveillance. GIS was utilized to map features such as administrative divisions, water bodies and land use patterns to identify potential vector breeding sites. Data on population statistics, road networks, schools and public and private health care providers were superimposed on topographical data. Geographic locations of identified malaria cases were entered in to the system to help in follow up. This database was maintained and verified on a monthly basis providing time trends of all collated information. Data management was carried out at the data monitoring station established at the Faculty of Medicine, University of Kelaniya (UoK).

A web-based, case based health information system (HIS) was developed to track surveillance activities. The surveillance model used was the same as that used by the AMC, the difference being that it was carried out by TEDHA. Its unique features in comparison to the AMC were the use of information technology in reporting data for monitoring purposes and creating a case based database. Details of all persons tested were entered and a mobile phone notification system was established to transmit SMS data from the field on a daily basis, two advanced features that the AMC surveillance system did not possess. The number of blood smears examined at each site, the number of positive slides and details of positive cases were transmitted from the field to the TEDHA data managers at the end of each working day. All parasitological and entomological data were stored in the HIS. Monthly, quarterly and annual reports were generated to assist routine monitoring and evaluation activities. These data were shared routinely with Regional Malaria Officers and AMC headquarters to facilitate rapid and effective responses. A newsletter summarizing the programme status was circulated bi-weekly via email nationally, regionally and internationally and periodic comprehensive reports were sent to the Global Fund and the Local Fund Agent (LFA).

### Collaborations with other agencies

All surveillance activities of TEDHA were planned in collaboration with the AMC. In addition, partnerships were developed with the armed forces stationed in the district for malaria surveillance. Periodic reviews were held with AMC and other funding bodies. Collaboration with universities ensured professional support for surveillance as well as in-service training and research opportunities for TEDHA employees.

### Monitoring and evaluation

Monitoring and evaluation of the programme was based on a framework stipulated by the donor agency (GFATM) and aligned with the National Malaria Strategic Plan and the National Malaria Programme Monitoring and Evaluation Plan. The framework provided indicators measuring the impact of the programme and the outcomes and outputs of the interventions. Data were routinely audited by the Local Fund Agent of the Global Fund as funding was based on performance. The audits included cross checking of all reported data including completeness, accuracy and timeliness. In addition the LFA employed their own consultant to assess the technical aspects of the programme.

Among the beneficiaries and stakeholders was the Government of Sri Lanka as well as the population in the areas where TEDHA was operating. Since population movement and cross border movement was high in that period the entire population of the districts was considered as the population at risk. The armed forces, school children, general public, religious leaders and community leaders provided a stakeholder framework in the five districts for inter-sectoral collaboration. The stakeholder framework supported the mobile malaria clinics in the community, entomological and vector surveillance in the districts and creating awareness among high risk groups and the community at large. Community mobilization teams were formed to liaise with stakeholders. Stake holder feedback was assessed as part of the logical framework.

## Results

### Establishment of district offices, malaria diagnostic laboratories and entomological surveillance sites

**Fig. 3:**
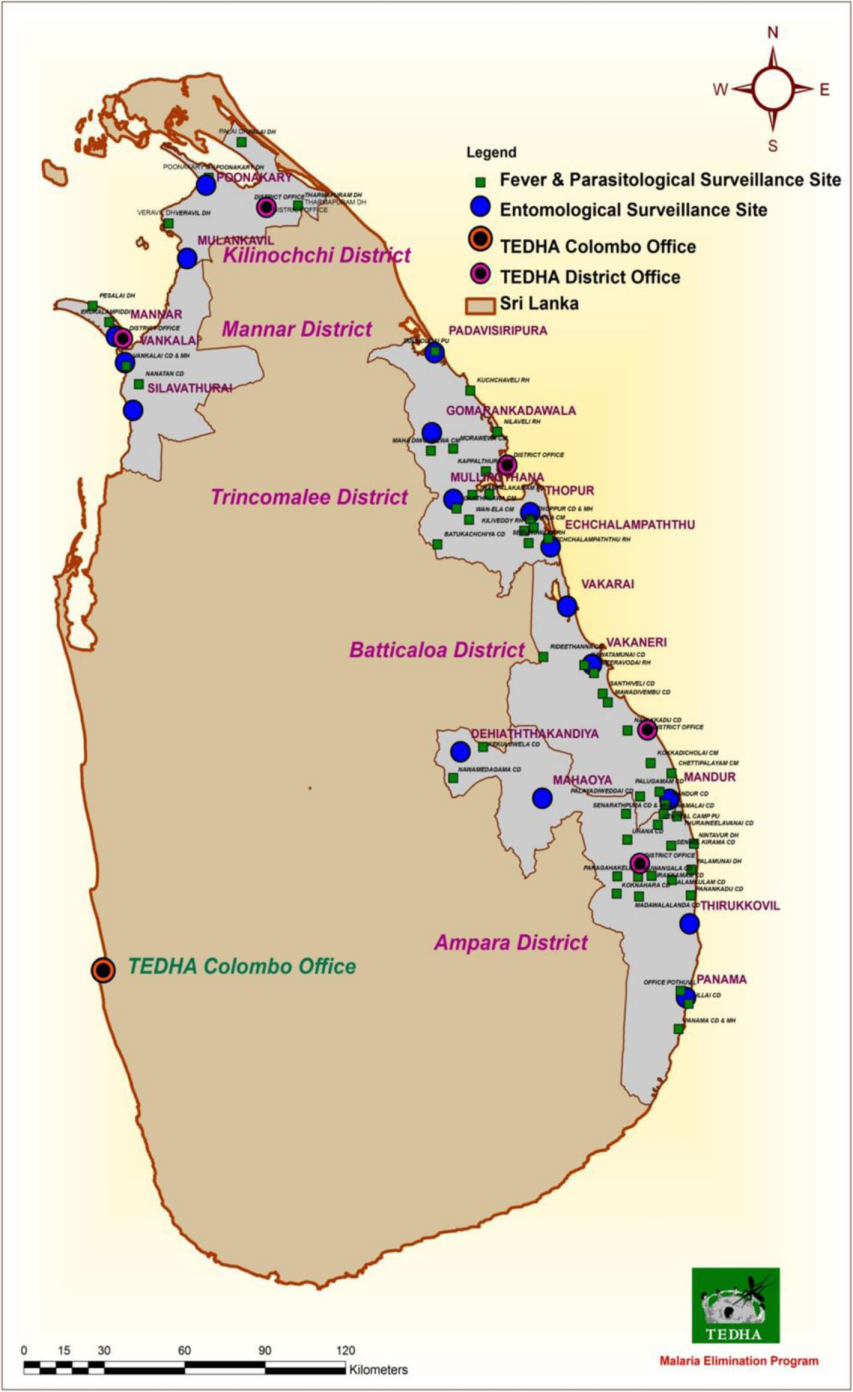
Distribution of TEDHA Offices, Malaria Diagnostic Laboratories in government hospitals and Entomological Surveillance sites

TEDHA operated in five war affected districts (out of 8) of Sri Lanka in two provinces covering 20% of the area of the country and approximately 9% of its population [9]. Many areas of these districts had not been cleared of landmines after the separatist war when TEDHA started operations. Figure 3 shows the distribution of TEDHA district offices, MDLs in government hospitals and entomological surveillance sites.

### Fever and parasitological surveillance

Fifty MDLs were established within hospitals in rural areas in the five districts by TEDHA and 131 individuals (including 19 supervising officers) were trained to carry out malaria microscopy. In addition, six MDLs were established in each of the district offices and in the sub-district office in the Ampara district. Each MDL was staffed with a fever surveillance assistant who prepared the blood smears (a role similar to the public health field officer of the AMC) and a parasitological surveillance assistant who stained, examined and reported on the blood smears (a role similar to the public health laboratory technician of the AMC). Parasitological surveillance began in phases from 2010 to 2012 in all the districts. During the period of operation, TEDHA screened 994,448 individuals (47.4%) for malaria from the five districts (Table 1). Of these, 243,867 were screened at MMCs. During this same period the AMC screened 1,102,054 individuals in the same districts. In the districts of Trincomalee and Ampara, TEDHA screened more blood smears for malaria than the AMC (Table 1). Nine cases of malaria (vivax malaria: 8, mixed: 1) were diagnosed by TEDHA, eight cases by ACD at MMCs in the Mannar district and one (possibly a relapse) was diagnosed by APCD in the Ampara district [6]. The AMC diagnosed 248 malaria cases from these districts in the same period. Of these, 145 cases were diagnosed from the Kilinochchi district prior to the commencement of TEDHA operations in that district (Table 2).

**Table 1:** Number of blood smears screened for malaria by TEDHA and the Anti Malaria Campaign 2010–2014 in the five districts

Year	Ampara		Trincomalee		Batticaloa		Mannar		Kilinochchi		Total	
	TEDHA (N) (%)	AMC (N) (%)	TEDHA (N) (%)	AMC (N) (%)	TEDHA (N) (%)	AMC (N) (%)	TEDHA (N) (%)	AMC (N) (%)	TEDHA (N) (%)	AMC (N) (%)	TEDHA (N) (%)	AMC (N) (%)
2010	34,028 (59.9)	22,784 (40.1)	24,151 (24.2)	75,535 (75.8)	25,218 (25.5)	73,849 (74.5)	25,305 (55.3)	20,484 (44.7)	0 (0%)	10,975 (100.0)	97,672 (32.4)	203,627 (67.6)
2011	108,862 (79.9)	27,343 (20.1)	70,096 (49.9)	70,330 (50.1)	50,547 (37.7)	83,458 (62.3)	18,989 (44.5)	23,716 (55.5)	0 (0%)	42,260 (100.0)	248,494 (50.1)	247,107 (49.9)
2012	80,738 (74.8)	27,160 (25.2)	84,432 (64.8)	45,775 (35.2)	49,168 (40.4)	72,498 (59.6)	21,014 (42.9)	27,992 (57.1)	2563 (5.3)	45,852 (94.7)	237,915 (52.0)	219,277 (48.0)
2013	89,033 (79.6)	22,834 (20.4)	76,866 (63.6)	43,988 (36.4)	56,160 (25.5)	78,208 (74.5)	15,933 (30.8)	35,755 (69.2)	9928 (18.7)	43,156 (81.4)	247,920 (52.5)	223,941 (47.5)
2014	52,766 (71.2)	21,336 (28.8)	53,659 (61.7)	33,333 (38.3)	35,738 (37.7)	60,212 (62.3)	10,536 (18.0)	48,062 (82.0)	9798 (17.8)	45,159 (82.2)	162,497 (43.8)	208,102 (56.2)
Total	365,427 (75.1)	121,457 (24.9)	309,204 (53.5)	268,961 (46.5)	216,831 (40.4)	368,225 (59.6)	91,777 (37.0)	156,009 (63.0)	22,289 (10.6)	187,402 (89.4)	994,498 (47.4)	1,102,054 (52.6)

**Table 2:** Number of indigenous malaria cases diagnosed by AMC and TEDHA in the five districts

Year	Ampara		Trincomalee		Batticaloa		Mannar		Kilinochchi		Total malaria cases reported from the five districts		Total indigenous malaria cases reported in the country
	AMC	TEDHA	AMC	TEDHA	AMC	TEDHA	AMC	TEDHA	AMC	TEDHA	AMC	TEDHA	
2010	9	0	6	0	2	0	101	08	88	–	206	8	684
2011	0	01	2	0	0	0	23	0	13	–	38	1	124
2012	0	0	1	0	0	0	1	0	2	0	04	0	23
2013	0	0	0	0	0	0	0	0	0	0	0	0	0
2014	0	0	0	0	0	0	0	0	0	0	0	0	0

### Entomological surveillance

Seventeen sentinel sites (4 localities per sentinel site, giving a total of 68 localities) were identified in the five districts. Seventeen entomological teams were recruited (136 technical staff and 7 supervising officers) from among local residents. From 2010 to 2014, TEDHA carried out 13,014 entomological surveillance days. A total of 13,660 traps (cattle baited hut traps and cattle baited net traps) were used to assess vector abundance and distribution of malaria vectors, and its association with climatic factors. A summary of results of entomological surveillance activities carried out is shown in Table 3.

**Table 3:** TEDHA entomological surveillance results; cattle baited hut/net collections and larval densities (July 2010–July 2014

	Cattle baited Hut collections			Cattle baited net collections			Number of larvae per 100 dips (number of larvae collected)	
	No of traps	No collected (average number of mosquitoes per trap)		No of traps	No collected (average number of mosquitoes per trap)		*An. culicifacies*	*An. subpictus*
		*A.culicifacies*	*A. subpictus*		*An.culicifacies*	*An. subpictus*		
Mannar	549	0 (0)	3957 (7.20)	1024	0 (0)	2079 (2.03)	0	19.37 (*n* = 45,522)
Kilinochchi	113	0	26.0 (0.230)	762	0 (0)	4660 (6.11)	0	5.7 (*n* = 10,980)
Ampara	1642	85 (0.051)	8895 (5.41)	2553	50 (0.09)	15,940 (6.24)	0.004 (*n* = 62)	0.141 (*n* = 899)
Batticaloa	1287	1 (0.0007)	5375 (4.17)	1791	2 (0.001)	15,559 (8.68)	0	2.05 (*n* = 8457)
Trincomalee	1995	112 (0.056)	5786 (2.90)	1944	8 (0.004)	3182 (1.64)	0.17 (*n* = 1074)	1.12 (*n* = 1829)

### Dataflow, management and follow up of malaria positives cases

All nine malaria positive cases were immediately notified to regional malaria officers and AMC after diagnosis. Follow up and screening of all patients was carried out by TEDHA in collaboration with the AMC. The data entered in the GIS was archived in the TEDHA database. Relevant information was shared with the AMC and other government organizations as and when requested. The summary statistics shown in tables were generated from the data recorded in the HIS. The data are now in possession of the AMC.

## Discussion

### Main findings of this study

This manuscript describes a case study where TEDHA, a private sector institution, was invited in to a partnership with the National Anti Malaria Campaign of Sri Lanka during 2010–2014 in war affected districts in Sri Lanka to enhance the malaria elimination efforts. This period was a critical time when the government health facilities were severely undermined in areas affected by the separatist war. The experience of TEDHA in Sri Lanka is a good example of how public-private partnerships can be utilized for better health outcomes [10] under special circumstances. After 5 years of operation, TEDHA successfully completed its operations in August 2014. Intermediate reports of activities carried out by TEDHA have been published earlier [7, 11].

The last case of indigenous malaria in Sri Lanka was reported in October 2012 [12]. Apart from the occasional cases of imported malaria reported since then, Sri Lanka has been certified as “malaria-free” by the WHO. Most cases of malaria between 2002 and 2012 were reported from provinces in which TEDHA was operating [13]. TEDHA contributed significantly as the large number of people screened ensured a high annual blood examination rate (ABER) and vulnerable people were screened in receptive areas, two important considerations for certification of malaria-free status. The extensive entomological surveillance activities carried out during the period monitored receptivity in these areas providing AMC with data for necessary response. TEDHA was able to fulfill targets set by the Ministry of Health and the Global Fund.

The TEDHA-AMC partnership was funded by the Global Fund. By the end of operations in August 2014, TEDHA had achieved all targets of the Global Fund performance framework. TEDHA achieved an A2 overall grade that indicated that Global Fund expectations were met after careful scrutiny [12].

The sharing of information and the collaboration in synchronized malaria control activities between the private and public sector institutions is an example of how partnerships can be developed for malaria elimination. All activities within TEDHA’s mandate were accomplished with the concurrence of the AMC and the Country Coordinating Mechanism of Sri Lanka (CCMSL). TEDHA enjoyed observer status in the CCMSL over the 5 year period from October 2009 till August 2014. TEDHA participated in the monthly review meetings of the Regional Malaria Officers (RMOs) conducted by the AMC at the AMC HQ in Colombo which facilitated data sharing and smooth operations. The infrastructure established by TEDHA through this programme remains functional and its facilities (MDLs established in rural hospitals) are now used by the institutions of the Ministry of Health, some for malaria diagnosis. Two TEDHA recruits completed Master in Philosophy (MPhil) and Doctor of Philosophy (PhD) degrees from the UoK, Sri Lanka based on their research work conducted while working for TEDHA.

### What is already known on the topic?

Public-private partnerships have been used in malaria control efforts previously with variable success. In Uganda and Kenya, the pharmaceutical company, Glaxo started a pilot programme in 1997 by donating up to one million treatment courses of malarone in what was subsequently described as the malarone donation programme [13]. The programme made an initial positive impact in pilot studies but later the project was deemed unsustainable. Tanzania had a more successful programme with the government offering subsidy vouchers to mothers at antenatal clinics to buy an insecticide treated bed net after paying a top-up fee [14]. An analysis of voucher redemption data from 2007 to 2011 showed that the programme had more than an 80% redemption rate especially after top up prices were reduced in 2009. The success of the campaign was probably due to involving a target group who were more likely to comply with advice. There are many other examples of similar partnerships which have aided malaria control efforts of different countries [15–18], some of which have been large investments with ambitious goals such as the Innovative Vector Control Consortium that focused on developing new pesticides for vector control [19]. Each country or region has to decide on the best strategies to utilize the available resources in such partnerships for maximum benefits for malaria control/elimination [19]. In Malaysia, the government successfuly secured the support of private sector plantations in malaria control efforts. Clinicians work with malaria control programme officers in private on-site clinics in the plantation sector in Sabah, Malaysia to treat malaria patients [20]. Some of the plantations are in the process of procuring microscopes for malaria diagnosis. A public-private partnership project similar to TEDHA’s activities has been recently initiated in Madhya Pradesh, India. In this project, the public-private partnership stakeholders jointly undertake malaria surveillance and elimination efforts by setting up management and technical committees [21].

### What this study adds

This study describes a case study in which the private sector complemented activities of the national malaria control programme in Sri Lanka in achieving malaria elimination by intensifying surveillance in an area where the health system was in disarray.

Attempts to measure cost-effectiveness of this programme in terms of the number of malaria infections detected in an elimination setting are inherently flawed. In a malaria elimination programme, the goal is to detect all infections till the last one, and radically treat them. It is not concerned with the number of cases detected and reported which is the focus of a control programme. The analogy that best describes the need to maintain a quality assured surveillance system in malaria elimination settings is the global polio eradication initiative. Even though wild polio-virus infections have been reported in only three countries in 2016, polio immunization is carried out throughout the world and cannot be discontinued until eradication is achieved [22]. Even though Sri Lanka is certified as “malaria-free”, diagnostic and treatment services and monitoring of receptivity will have to continue at whatever cost.

We can identify some key factors that led to the success of this partnership. Firstly, many employees in surveillance teams were recruited locally from areas in which TEDHA operations were conducted; they were conversant in the local language and were known to the community. Despite some of them not having technical expertise beforehand, it was decided to recruit and train them with the expectation of smooth transition in the long term. Secondly, engagement and education of local communities was advantageous to TEDHA operations. TEDHA invested on recruiting community mobilization officers from local communities. Access to entomological surveillance localities was easier and safer when local workers were included in the teams. Thirdly, TEDHA concentrated on capacity building and sustainable development. Many hospitals were developed with laboratory facilities and access to running water and electricity were re-established. TEDHA provided an useful platform to develop these institutions further with government funding which became available later. Two of TEDHA associates obtained postgraduate qualifications by research while working for the programme. Fourthly, TEDHA employed highly skilled professionals for managerial tasks and scientific tasks (surveillance, quality control) at executive level. Fifthly, TEDHA maintained ongoing close collaborations and discussions with all government and non-governmental stakeholders such as funding agencies, Ministry of Health, AMC and higher education institutions such as the Universities of Colombo, Kelaniya and Sri Jayewardenepura.

The model described here highlights the feasibility of engaging the private sector in malaria elimination settings when the health system is weak. During the time TEDHA operations were in progress, the government strengthened the health system in the previously war torn areas. The Global Fund assisted this process with a grant of USD 7.8 million in 2010 for health systems strengthening [12]. Additional infrastructure was provided and staff was trained. When TEDHA completed its work, there was a smooth transition of operations to the health system in these areas which was similar to that in other areas of the country.

### Limitations

There were some initial problems in coordinating AMC and TEDHA activities and in amalgamating AMC and TEDHA data due to concerns about propriety and the nature of collaboration as this was the first time that the Ministry of Health was engaged in such an enterprise. These initial hiccups were sorted out with the development of a common monitoring and evaluation plan, after which operations of the AMC and TEDHA were coordinated with agreement from both AMC and TEDHA.

## Conclusion

We report a case study of a successful public-private partnership model that was implemented in a country that has been devastated by malaria for centuries in a malaria elimination programme which was initiated at a time when the health system was in disarray in some parts of the country due to an armed conflict. We highlight here the feasibility of using such a model even in the short term to achieve the goal of malaria elimination. This model may be used in settings in other countries in the region and beyond which are targeting malaria elimination in the near future.

## Abbreviations

ACD: Active case detection
AMC HQ: Anti Malaria Campaign Headquarters
AMC: Anti Malaria Campaign
APCD: Activated passive case detection
CCMSL: Country Coordinating Mechanism Sri Lanka
DO: District office
GFAMT: Global Fund Against AIDS, Tuberculosis and Malaria
GIS: Geographical information system
HIS: Health information system
LFA: Local Funding Agent
MDL: Malaria diagnostic laboratory
OPD: Outpatients Department
PCD: Passive case detection
RMO: Regional Malaria Officer
TEDHA: Tropical and Environmental Diseases and Health Associates
UoK: University of Kelaniya
WHO: World Health Organization
